# Study of Incidence of Gross Urogenital Lesions and Abnormalities on Does Slaughtered at Nyagatare Slaughterhouse, Eastern Province, Rwanda

**DOI:** 10.1155/2017/7564019

**Published:** 2017-12-03

**Authors:** Borden Mushonga, Sylvine Twiyizeyimna, Gervais Habarugira, Erick Kandiwa, Simbarashe Chinyoka, Alaster Samkange, Alec Bishi

**Affiliations:** ^1^School of Veterinary Medicine, Faculty of Agriculture and Natural Resources, University of Namibia, P. Bag 13301, Pioneerspark, Windhoek, Namibia; ^2^School of Animal Sciences and Veterinary Medicine, College of Agriculture, Animal Sciences and Veterinary Sciences, University of Rwanda, P.O. Box 57, Nyagatare, Rwanda

## Abstract

Reproductive and urinary tract abnormalities are a cause of infertility, reproductive inefficiency, and economic losses in goats. The aim of this study was to investigate the occurrence and nature of reproductive and urinary tract abnormalities encountered in female goats slaughtered at Nyagatare abattoir in the Eastern Province of Rwanda. Reproductive and urinary organs from 369 female goat carcasses were opened by incision and then given a thorough macroscopic examination by visually inspecting and palpating for evidence of abnormalities. The results showed that there was an overall occurrence of 7.8% reproductive organ/tract abnormalities and 10.6% urinary organ/tract abnormalities. Ovarian hypoplasia was the reproductive abnormality with the highest overall occurrence (32.3%) and renal calculi were the urinary organ abnormality with the highest occurrence (38.1%). 95.2% of the reproductive organ/tract abnormalities observed usually result in infertility and 91.3% of the urinary organ/tract abnormalities observed result in economic losses through condemnation of kidneys at slaughter. The high incidence of the observed urinary organ/tract abnormalities represents a potential public health challenge. There was no significant difference in the occurrence of reproductive organ/tract abnormalities according to breed (*p* > 0.05, *n* = 31). There was also no significant difference in the occurrence of urinary organ abnormalities according to breed (*p* > 0.05, *n* = 42).

## 1. Introduction

With a population of 11.3 million people in 2013, Rwanda had one of the highest human population densities in Africa with 445 ppe/km^2^ [1]. Expansion of large ruminant production would exacerbate this land deficit. Small ruminants (sheep and goats), however, offer a flexible production system that does not require extensive tracts of land [2]. Within the African society goats usually form a greater proportion of the total wealth of poor families because of the low input and low maintenance requirements using small pieces of marginal lands and poor pasture [3]. Furthermore, goats have been recommended as an efficient production system for Rwanda [4].

In a few years, the goat population in Rwanda had grown to 2.97 million by 2010, 6.7% of which were in the Eastern Province [1]. Goat meat contributed 13.5% of the total meat produced in Rwanda in 2011 [1]. The main breeds of goat found in Rwanda are the Small East African Goat and its crosses with the Alpine and the Anglo-Nubian goat [5]. Each year a significant loss in goat numbers results from death of animals, inferior weight gain, and male and female infertility resulting in reproductive inefficiency [6].

Male and female infertility result in reduced productivity in goats. A recent publication [7] of male genital abnormalities in cattle at Nyagatare abattoir reported that about 30% of slaughtered bulls showed reproductive abnormalities of one form or the other. It has previously been argued that reproductive and urinary tract abnormalities may cause pain which in turn may have a negative effect on mating behaviour in livestock [8].

Pathology of the female reproductive tract affects the productivity of a livestock enterprise because it affects the fertility of the herd or flock [9] and most of this information has, historically, been obtained through abattoir surveys [9–11]. There are a number of studies on the prevalence of several abnormalities of the female reproductive tract of sheep [9, 10] and goats [11–14]. Most of the reports on female reproductive abnormalities emanate from the Middle East [10, 11, 15–18], the Indian subcontinent [13, 14, 19, 20], and sub-Saharan Africa [12, 20, 21].

The prevalence of female reproductive abnormalities in different breeds of sheep reportedly vary from 0.72% to as much as 52.7% [10, 15, 18] whilst that in goats vary from 8.08% to 38% [11, 18, 19, 22–24]. Female reproductive tract pathology predominantly affects the uterus, followed by the ovary, the cervix, fallopian tubes, and vagina in that order [2, 6] mainly due to parasites such as trypanosomes [25, 26] and environmental pollutants such as arsenic [27] and neoplasia [6, 17].

Gross disorders of the urinary system are not common in abattoir studies. About 6.8% of the kidneys were condemned for nephritis in apparently healthy sheep and goats at the Helmex abattoir in Ethiopia [28]. Hydronephrosis can be congenital or acquired as a result of urinary tract obstruction [29]. Pyelonephritis, renal infarction, and necrosis are usual sequela to hydronephrosis though they can also result from ascending* Corynebacterium renale* or* Escherichia coli* infections of the urinary tract [30]. Urinary calculi in animals result from precipitation of dietary calcium oxalate in urine leading to painful conditions like nephritis and cystitis.* Tribulus terrestris* was reported as one of the toxic causes of renal necrosis and renal calculi in goats [31].

To the best of our knowledge, there is no published abattoir information on reproductive abnormalities in female that reported the prevalence of male reproductive abnormalities in cattle in Nyagatare. In Rwanda, only a handful of studies on reproductive abnormalities in dairy [32, 33] and beef cattle [34–36] are available. This study aimed to identify and measure the period prevalence of reproductive organ and urinary tract abnormalities in female goats slaughtered at Nyagatare abattoir between March and June 2013. The study also aimed to find the correlation between the breed of goat and the abnormalities encountered.

## 2. Materials and Methods

### 2.1. Setting

The study was conducted in Nyagatare, one of the seven districts of the Eastern Province of Rwanda. The district is located at 01°18′S and 30°20′E. The average altitude is 1513.5 m above sea level.

### 2.2. Materials

A total of 396 does slaughtered at Nyagatare abattoir were used in this study. The goat production system is extensive herding/browsing and night kraaling. According to abattoir records 299 of the does were of the indigenous (local) breed and 97 were cross breeds.

### 2.3. Design

A cross-sectional study was carried out at Nyagatare abattoir in the Eastern Province, Rwanda. The study was conducted over a period of four months from March to June 2013. The sample consisted of all does aged 6 months or older that came to the abattoir during the period of the study.

### 2.4. Procedure

After slaughter, the relative anatomical configurations of the reproductive organs and urogenital tracts were assessed in situ. They were then carefully removed intact. Abnormalities in shape, size, or colour were noted and recorded. The samples were then placed in sterilized containers for transportation to Umtara Veterinary Laboratory for further examination. At the laboratory, each reproductive tract was cut open from the vulva, through the vagina, past the cervix and uterine body into each uterine horn up to the ovaries. The urinary bladder and both kidneys were also cut open. A thorough macroscopic examination by visual inspection and palpation was performed for the identification of the colour, size, shape, and consistency of any lesions found on the reproductive organs and urogenital tracts. The findings of the macroscopic examination were used as the basis for the diagnosis of the various abnormalities observed.

### 2.5. Analyses

Descriptive statistics were used for the analysis of the findings. Categorical variables were described using percentages whilst bivariate analysis was performed using chi-square and Fischer's exact tests. Collected data were entered and managed in MS Excel and Statistical Package for Social Sciences (SPSS) version 16.0. The *Z* test for comparison of proportions was used and *p* values ≤ 0.05 were considered significant.

## 3. Results

### 3.1. Overall Occurrence of Abnormalities

**Figure 1:**
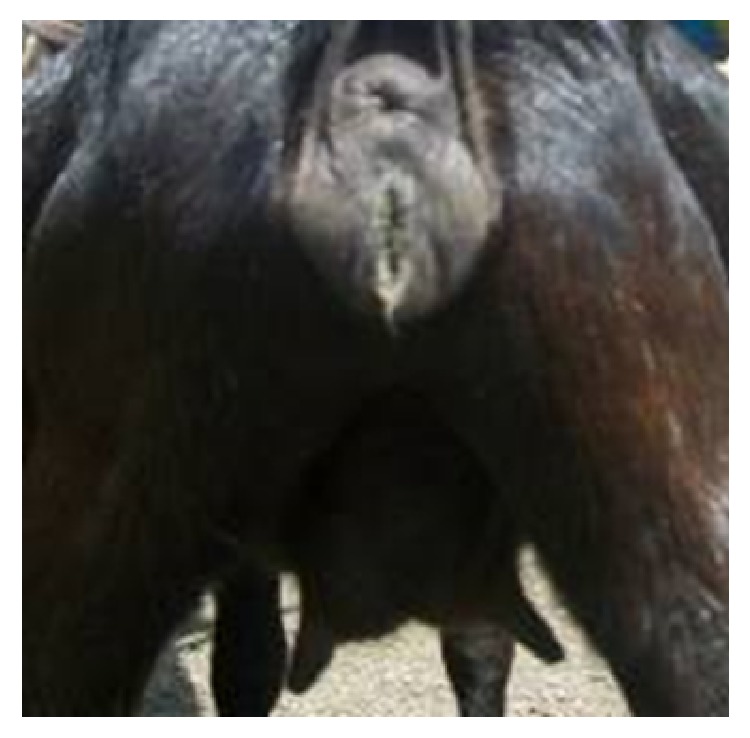
Vulvovaginitis in a doe.

During the 4 months of the study, 7.8% (*n* = 396) of the 396 female goats (does) were slaughtered at Nyagatare abattoir and examined for abnormalities, and 31 had reproductive organ/tract abnormalities (Table 3). The types of abnormalities observed were ovarian hypoplasia, ovarian cysts, endometritis, pyometra, hydrometra, mummified foetus, salpingitis, haematosalpinx, cervical atresia, vaginitis, and granular vulvovaginitis (Figure 1). The most commonly observed abnormality was ovarian hypoplasia which occurred in 32.3% (*n* = 31) of the goats with reproductive organ/tract abnormalities (Table 1). 91.3% of the reproductive organ/tract abnormalities (exclusive of vaginitis and vulvovaginitis) are usually a direct cause of infertility in female livestock. Hypoplasia was defined in this study as an ovary that was less than 0.5 cm diameter without grossly visible corpora lutea or follicles in an animal that was older than 8 months [37].

As indicated in Table 4, 10.6% (*n* = 396) of the 396 goats examined had urinary organ/tract abnormalities. The types of abnormalities observed were renal calculi, hydronephrosis, pyelonephritis, renal abscess, renal infarction, renal necrosis, and cystitis. The most commonly observed urinary organ abnormality was renal calculi which occurred in 38.1% (*n* = 42) of the goats with urinary organ abnormalities (Table 2). 95.2% of urinary organ/tract abnormalities (excluding cystitis) all result in condemnation of the kidneys at slaughter.

The occurrence of reproductive organ/tract abnormalities was higher (35%) in cross breed goats (Table 5) and lower (27.3%) in indigenous goats (Table 6). There was no significant difference in the occurrence of urinary organ abnormalities according to breed (*p* > 0.05).

The occurrence of urinary organ/tract abnormalities was higher (41.4%) in indigenous goats (Table 7) and lower (30.8%) in cross breed goats (Table 8). There was no significant difference in the occurrence of urinary organ abnormalities according to breed (*p* > 0.05).

## 4. Ethical Considerations

Ethical approval (by official confirmation notice) for this study protocol was obtained from the Institutional Review Board of the School of Animal Sciences and Veterinary Medicine, College of Agriculture and Veterinary Sciences, University of Rwanda. The reproductive and urinary tract examination procedures were performed by a qualified veterinary pathologist assisted by meat inspectors through routine ante- and postmortem inspection aimed at ensuring personnel safety. Pathological lesions were differentiated and judged based on Herenda et al. (1994) guidelines on meat inspection for developing countries [38]. The abattoir authorities were informed about the study purpose and procedures and provided written consent prior to commencement of the study.

## 5. Discussion

This study revealed a 7.8% overall occurrence of reproductive organ/tract abnormalities which consisted of ovarian hypoplasia, cystic ovaries, endometritis, pyometra, hydrometra, haematosalpinx, salpingitis, cervical atresia, vaginitis, and vulvovaginitis in does slaughtered at Nyagatare abattoir in the Eastern Province of Rwanda. This figure is nearly identical to the findings of other authors [18] who reported a prevalence of 7.11% and 8.08% uterine abnormalities in sheep and goats, respectively, and almost similar with findings by other workers who reported a prevalence of 10.11% [39].

The study also revealed a 10.6% overall occurrence of urinary organ/tract abnormalities which consisted of renal calculi, renal infarction, renal necrosis, renal abscess, pyelonephritis, hydronephrosis, and cystitis. These findings are nearly double the 6.4% prevalence reported by other workers [28]. The figure is many folds over the 0.05% nephritis reported elsewhere [20]. The case of the latter report is understandable as our study considered the whole urinary system whereas former study only recorded kidney afflictions [20].

Studies in the Sahel region of Nigeria reported that 17.88% of the slaughtered goats between 1998 and 2009 were pregnant [40]. The period during which the current study was carried out (March to June) was not necessarily within the gestational season of goats in tropical Africa and this explains the near absence of pregnant animals at slaughter. A longer study encompassing the gestational season of the goats may reveal whether there are proper selection and exclusion of pregnant goats from those destined for slaughter.

The importance of this study is that it established the presence of reproductive and urinary organ/tract abnormalities in otherwise healthy female goats. The findings were made in spite of the fact that Rwanda had the highest growth rate (9.6% per annum) in goat meat production in the whole of the East African region [1]. Were it not for the prevalence of these genital abnormalities, the growth rate of goat production would have been even higher.

Inadequate nutrition was a possible cause of the high occurrence of ovarian hypoplasia observed in this study. Infertility directly results from ovarian hypoplasia. Nutritional deficiency and low energy diets were some of the major causes of inactive ovaries observed in goats during the postpartum period [41]. Work in East African indigenous goats showed that ovarian hypoplasia led to decreased oestrogen levels and irregular oestrus cycles as a result of artificially induced cobalt deficiency [42].

The prevalence of endometritis, pyometra, and hydrometra in this study was higher than that from other studies [21]. In cases of hydrometra, accumulation of foetal fluids after embryonic death may result in pseudopregnancy rendering the doe infertile for that duration [12, 43].

The 27.3% prevalence of ovarian hypoplasia from the current study was much higher than the 1% reported in sheep [9] and was not associated with* uterus unicornis*. The overall prevalence of ovarian cysts in this study (6.5%) was similar to that in a study in culled ewes [11] though it was higher than that by other workers [10, 21]. Ovarian cysts are a direct cause of infertility in animals [44]. Trypanosomiasis, a disease that has been mentioned to occur in Rwanda [45], is a common cause of infertility of ovarian cyst origin in tropical African ruminants, horses, and rodents [25] and may have been responsible for observed ovarian cysts.

Hydrosalpinx is considered to be an irreversible stage of fallopian tube inflammatory conditions (salpingitis) and can render an animal sterile. These inflammatory conditions may result from invasion of infectious organisms. The overall prevalence of salpingitis and haematosalpinx in this study (13%) is higher than that from other studies [10]; variation in prevalence from other studies could be attributed to different animal production systems and nutritional conditions that vary from one location to another. It is also possible that a longer study with more animals may result in lower prevalence of some of the abnormalities.

This study did not, however, take into consideration the age of animals at slaughter as done by other workers who proved that susceptibility to reproductive disease increases with age [21].

This study also revealed a 10.6% overall occurrence of urinary organ/tract abnormalities 91.3% of which were associated with the kidneys. The urinary organ/tract abnormalities consisted of renal calculi, hydronephrosis, pyelonephritis, cystitis, renal infarction, necrosis, and abscess. These findings implicated sizeable financial losses through condemnation of kidneys at slaughter. They also show the presence of a potential public health hazard in situations where goats not showing obvious signs of disease are slaughtered outside abattoirs for human consumption. Though these results were obtained from female goats, there is a high likelihood that the same urinary organ/tract exist in the male goats of the Eastern Province of Rwanda. Such an occurrence would result in reduced mating behaviour as a result of the pain associated with these abnormalities ultimately resulting in subfertility of goat flocks.

Results from this study showed the lack of correlation between breed of goat and the occurrence of reproductive and urinary organ/tract abnormalities in the female goats slaughtered at Nyagatare. The fact that study period did not coincide with the gestational season of goats in Rwanda explains why only one pregnant animal (a case of mummified foetus) was recorded. Designation of a wider study period encompassing the gestational season of goats may be necessary to ascertain whether there is wastage due to slaughter of pregnant animals comparable to those found elsewhere in the region [40].

## 6. Conclusion

The findings of this study showed an overall prevalence of 7.8% for female reproductive organ/tract abnormalities 91.3% of which have a direct negative effect on fertility thereby presenting a problem to growth of goat production in Eastern Rwanda. The fact that Rwanda had the fastest growth rate of goat populations of 9.6% per annum in the East African region, however, implies that these reproductive abnormalities are an even worse problem in the rest of the region or that there are other environmental, human, and/or animal factors limiting the growth rate of goat production, all of which would require separate studies. The findings of this study reveal a 10.6% prevalence for female urinary organ/tract abnormalities 95.2% of which were of the kidneys which implicates notable economic losses due to condemnation of edible organs and an obvious public health threat from consumption of diseased organs in goats slaughtered outside the abattoir system. Urinary organ/tract abnormalities documented in this study may also indirectly result in increased infertility in goat flocks as the pain associated with these conditions negatively impacts on mating behaviour. This study will provide basic knowledge on the reproductive and urogenital health of the female goats in Eastern Rwanda that can be used to guide further investigations throughout the country or in the implementation of effective treatment and control strategies aimed at reducing the incidence of doe infertility.

## Figures and Tables

**Table 1:** Overall occurrence of reproductive organ abnormalities in does.

Nature of abnormality	Frequency	Relative frequency (%)
Ovarian hypoplasia	10	32.3
Endometritis	5	16.1
Pyometra	5	16.1
Ovarian cysts	2	6.5
Salpingitis	2	6.5
Granular vulvovaginitis	2	6.5
Vaginitis	1	3.2
Haematosalpinx	1	3.2
Hydrometra	1	3.2
Mummified foetus	1	3.2
Cervical atresia	1	3.2
Vaginitis	1	3.2

*Total*	*31*	*-*

**Table 2:** Overall occurrence of urinary organ abnormalities in does.

Nature of abnormality	Frequency	Relative frequency (%)
Renal calculi	16	38.1
Hydronephrosis	10	23.8
Pyelonephritis	7	16.7
Renal abscess	4	9.5
Renal infarction	2	4.8
Cystitis	2	4.8
Renal necrosis	1	2.4

*Total*	*42*	*-*

**Table 3:** Occurrence of reproductive tract abnormalities according to breed of doe.

Breed	Number of slaughtered animals	Frequency of abnormalities	Relative frequency (%)
Indigenous	299	20	6.7
Cross	97	11	11.3

*Overall*	*396*	*31*	*7.8*

**Table 4:** Occurrence of urinary tract abnormalities according to breed of doe.

Breed	Number of slaughtered animals	Frequency of abnormalities	Relative frequency (%)
Indigenous	299	29	9.7
Cross	97	13	13.4

*Overall*	*396*	*42*	*10.6*

**Table 5:** Occurrence of reproductive organ/tract abnormalities in cross breed does.

Nature of abnormality	Frequency	Relative frequency (%)
Ovarian hypoplasia	7	35
Ovarian cysts	1	5
Haematosalpinx	1	5
Salpingitis	1	5
Endometritis	3	15
Pyometra	3	15
Hydrometra	1	5
Mummified foetus	0	0
Cervical atresia	1	5
Vaginitis	1	5
Granular vulvovaginitis	1	5

*Total*	*20*	*-*

**Table 6:** Occurrence of reproductive organ/tract abnormalities in indigenous does.

Nature of abnormality	Frequency	Relative frequency (%)
Ovarian hypoplasia	3	27.3
Ovarian cysts	1	9.1
Haematosalpinx	0	0
Salpingitis	1	9.1
Endometritis	2	18.2
Pyometra	2	18.2
Hydrometra	0	0
Mummified foetus	1	9.1
Cervical atresia	0	0
Vaginitis	0	0
Granular vulvovaginitis	1	9.1

*Total*	*11*	*-*

**Table 7:** Occurrence of urinary organ/tract abnormalities in indigenous does.

Nature of abnormality	Frequency	Relative frequency (%)
Renal calculi	12	41.4
Hydronephrosis	7	24.1
Pyelonephritis	5	17.2
Renal abscess	3	10.3
Renal necrosis	1	3.4
Cystitis	1	3.4
Renal infarction	0	0

*Total*	*29*	*-*

**Table 8:** Occurrence of urinary tract abnormalities in cross breed does.

Nature of abnormality	Frequency	Relative frequency (%)
Renal calculi	4	30.8
Hydronephrosis	3	23.1
Pyelonephritis	2	15.4
Renal infarction	2	15.4
Renal abscess	1	7.7
Cystitis	1	7.7
Renal necrosis	0	0

*Total*	*13*	*-*
